# Molecular genetics of microsatellite-unstable colorectal cancer for pathologists

**DOI:** 10.1186/s13000-017-0613-8

**Published:** 2017-03-04

**Authors:** Wei Chen, Benjamin J. Swanson, Wendy L. Frankel

**Affiliations:** 1 Department of Pathology, The Ohio State University Wexner Medical Center, S301 Rhodes Hall, 450 W. 10th Ave, Columbus, Ohio 43210 USA; 2 Department of Pathology, University of Nebraska Medical Center, 985900 Nebraska Medical Center, Omaha, NE 68198 USA; 3 Department of Pathology, The Ohio State University Wexner Medical Center, 129 Hamilton Hall, Columbus, Ohio 43210 USA

**Keywords:** Mismatch repair protein, MMR, Lynch syndrome, Microsatellite instability, MSI, Colorectal cancer, Molecular genetics, Immunohistochemistry

## Abstract

**Background:**

Microsatellite-unstable colorectal cancers (CRC) that are due to deficient DNA mismatch repair (dMMR) represent approximately 15% of all CRCs in the United States. These microsatellite-unstable CRCs represent a heterogenous group of diseases with distinct oncogenesis pathways. There are overlapping clinicopathologic features between some of these groups, but many important differences are present. Therefore, determination of the etiology for the dMMR is vital for proper patient management and follow-up.

**Main body:**

Epigenetic inactivation of *MLH1* MMR gene (sporadic microsatellite-unstable CRC) and germline mutation in an MMR gene (Lynch syndrome, LS) are the two most common mechanisms in the pathogenesis of microsatellite instability in CRC. However, in a subset of dMMR CRC cases that are identified by screening tests, no known LS-associated genetic alterations are appreciated by current genetic analysis. When the etiology for dMMR is unclear, it leads to patient anxiety and creates challenges for clinical management.

**Conclusion:**

It is critical to distinguish LS patients from other patients with tumors due to dMMR, so that the proper screening protocol can be employed for the patients and their families, with the goal to save lives while avoiding unnecessary anxiety and costs. This review summarizes the major pathogenesis pathways of dMMR CRCs, their clinicopathologic features, and practical screening suggestions. In addition, we include frequently asked questions for MMR immunohistochemistry interpretation.

## Background

Colorectal cancer (CRC) represents the third most common malignancy diagnosed both in men and women in the United States. Approximately 15% of CRC display a defect in the mismatch repair pathway [1], resulting in microsatellite instability (MSI). The identification of MSI CRC from microsatellite-stable (MSS) tumors is clinically important, because MSI tumors have a better stage-adjusted survival compared to MSS tumors and may respond differently to 5-fluorouracil-based adjuvant chemotherapy [2]. In addition, it is important to identify those patients with Lynch Syndrome (LS).

Defects in the mismatch repair (dMMR) mechanism leads to the pathogenesis of MSI tumors. The MMR mechanism identifies and fixes base-pair mismatches that occur within the genome. Proteins within the MMR system include MLH1, PMS2, MSH2, MSH6, MLH3, MSH3, PMS1, and Exo1. These proteins form heterodimers that repair DNA damage. The most common and relevant heterodimers in colorectal carcinogenesis are MLH1/PMS2 and MSH2/MSH6. Microsatellites, also known as short tandem repeats, are composed of mono- to hexa-nucleotides that constitute a repeated motif. These microsatellites constitute up to 3% of the genome and are variable in length. Chromosomal alleles often contain different lengths of the same microsatellite. Due to their repetitive nature, microsatellites are highly susceptible to errors in MMR; therefore dMMR tumors demonstrate microsatellite instability and are often hyper- or ultra-mutated [3].

MSI CRC identified by screening tests (MMR immunohistochemistry, IHC, or polymerase chain reaction, PCR) can be further divided into four categories after additional testing [4] (also see Table 1):

**Table 1:** Comparison of microsatellite-unstable colorectal cancers

	(A) Sporadic dMMR – *MLH1* Promoter Hypermethylation	(B) Lynch Syndrome	(C) Sporadic dMMR – Somatic MMR Mutations	(D) Constitutional Mismatch Repair Deficiency (CMMRD)
Germline mutation	None	One allele of a MMR gene (*MLH1, PMS2, MSH2, MSH6*)	None	Both alleles of a MMR gene (*MLH1, PMS2, MSH2, MSH6*)
Somatic mutation	*BRAF* V600E	2^nd^ allele of the mutated MMR gene	Both alleles of a MMR gene (*MLH1, PMS2, MSH2, MSH6*)	None
Epigenetic alteration	Somatic biallelic promoter methylation of *MLH1*	Germline deletion in 3′ end of *EPCAM* leads to *MSH2* methylation	None	None
Intense Lifelong Screening	No	Yes	No	Yes

*Abbreviations: dMMR* mismatch repair deficiency, *MMR* mismatch repair

*Adapted from Carethers and Stoffel* [4]

(A) Sporadic dMMR – *MLH1* promoter hypermethylation: MSI CRC due to hypermethylation of CpG islands in the *MLH1* promoter (*these tumors often arise via the serrated pathway, harbor BRAF mutation, and account for 10% to 15% of CRC*);
(B) Lynch Syndrome due to germline mutations in one of the MMR genes (*MLH1, PMS2, MSH2, MSH6*) or alteration in *EPCAM (TACSTD1)* gene that causes epigenetic silencing of *MSH2* [5] *(these tumors often arise in tubular adenomas and account for approximately 3% of CRC*);
(C) Unexplained dMMR Colorectal Cancers (sporadic dMMR-somatic MMR mutation and others): Cases with neither identified germline mutation of MMR nor hypermethylation of the promoter region of *MLH1 (these cases have unexplained dMMR and have been termed Lynch-like by some);*
(D) Rarely, constitutional MMR deficiency syndrome (*biallelic germline MMR mutations; of note, the normal adjacent tissue in these cases will also have dMMR*).

## Main text

### Sporadic dMMR colorectal cancers – *MLH1* promoter hypermethylation/CpG Island Methylator Phenotype (CIMP)

Tumors that show DNA hypermethylation at multiple promoters are classified as showing the CpG Island Methylator Phenotype (CIMP). There are several types of epigenetic alterations, including DNA hypermethylation, that regulate gene expression. Promoter methylation suppresses gene transcription by inhibiting binding of transcription factors, affecting histone acetylation, and altering conformations to effectively block access of transcriptional machinery to the gene. Epigenetic silencing in tumors is biologically equivalent to acquiring an inactivating mutation and may account for the first, second, or both hits in silencing tumor suppressor genes. Genes that are commonly hypermethylated in CRC include *MLH1*, *MCC* (methylated in CRC), *APC* and *MGMT*. The promoter region of five genes has been chosen as proxy markers of CIMP: *CACNA1G*, *IGF2*, *NEUROG1*, *RUNX3* and *SOCS1* [6]. Hypermethylation of at least three markers is the definition of CIMP. CRC with CIMP frequently harbor *BRAF* mutations and show methylation of *MLH1* resulting in MSI in up to 70% of cases. Of interest, the CDX2 negative/Cytokeratin 20 negative immunohistochemical phenotype defines a distinct subgroup of MSI CRCs with poor differentiation, CIMP status, and unfavorable prognosis [7].

### Lynch syndrome

LS is an autosomal dominant disorder that increases the risk of developing CRC and endometrial adenocarcinoma, as well as tumors of the small intestine, stomach, ureter, renal pelvis, ovary, brain, prostate [8], among others. LS is a result of deleterious germline mutations in the genes associated with DNA MMR (*MLH1*, *PMS2*, *MSH2*, *MSH6* and *EPCAM)*. Cancers (such as CRC) arise when a second hit occurs in the unaffected wild type allele, through various mechanisms such as loss of heterozygosity, mutation, or hypermethylation. Most (90%) CRC due to LS have MSI due to the defective MMR mechanisms caused by the germline mutation in association with the second hit. It is the most common hereditary CRC syndrome, with approximately 2–5% of all CRCs due to LS. Patients with LS benefit from increased surveillance; therefore, identification of patients as well as family members with this syndrome is very important. Historically, these cases were grouped with other familial CRC cases and referred to as Hereditary Nonpolyposis Colorectal Cancer (HNPCC). The term HNPCC is no longer preferred due to observations that patients with LS do have adenomatous polyps and these adenomatous polyps are more likely to progress to CRC [9].

The Amsterdam criteria were initially developed in 1990 [10] and later revised to Amsterdam II criteria in 1998 [11], to help identify families likely to have HNPCC for research purposes by using personal and family histories. The Amsterdam criteria include a series of clinical criteria that are also known as the “3-2-1” rule (Table 2). The Bethesda guidelines were developed in 1997 to identify patients who should have tumor screening for LS [12]. The revised Bethesda guidelines proposed in 2004 [13] incorporated histologic components into the screening guidelines. A comparison of the Amsterdam criteria and the revised Bethesda criteria is detailed in Table 2. Unfortunately, a significant portion of CRC patients with LS were still missed when using these criteria alone without MMR IHC or PCR testing [14]. Therefore, it is currently recommended that all newly diagnosed patients with CRC undergo LS screening by IHC and/or MSI PCR testing as recommended by many authoritative organizations, including the Evaluation of Genomic Applications in Practice and Prevention (a working group sponsored by the Centers for Disease Control and Prevention) in 2009 [15], the National Comprehensive Cancer Network in 2014 [16], the US Multi-Society Task Force in 2014 [17], the American College of Gastroenterology [18] and the American Society of Clinical Oncology [19] in 2015. Patients who have clinical features concerning for LS, but whose screening results indicate microsatellite stability, should be further evaluated by another technique such as MSI PCR (if initial screening was by IHC staining) or IHC for the MMR proteins (if initial screening was by MSI molecular testing), or possibly genetic sequencing of MMR genes since the sensitivity of the MSI by PCR and IHC is not 100%.

**Table 2:** Comparison of the Amsterdam criteria, Amsterdam II criteria, and the revised Bethesda criteria

Amsterdam criteria (meet all sub criteria)	Amsterdam II criteria (meet all sub criteria)	Revised Bethesda criteria (meet one of the following sub criteria)
**3** or more relatives with histologically confirmed colorectal cancer	**3** or more relatives with Lynch syndrome-associated cancer (colorectal cancer or cancer of the endometrium, small intestine, ureter or renal pelvis); cancers are histologically verified	Colorectal cancer diagnosed in a patient aged <50 years
**2** or more successive generations involved	**2** or more successive generations affected	Presence of synchronous, metachronous colorectal cancer or other Lynch syndrome-related tumors: cancer of the colorectum, stomach, small intestine, pancreas, biliary tract, renal pelvis, ureter, ovary, brain; sebaceous gland adenoma or carcinoma and keratoacanthoma, regardless of age
**1** or more of the cancers diagnosed before age 50 years	**1** or more relatives diagnosed before the age of 50 years	Colorectal cancer with MSI phenotype, especially lymphocyte infiltration, diagnosed in a patient aged <60 years
One should be a first-degree relative of the other two	One should be a first-degree relative of the other two	Patient with colorectal cancer and a first-degree relative with a Lynch syndrome-related tumor, with one of these cancers diagnosed at age <50 years
Familial adenomatous polyposis should be excluded	Familial adenomatous polyposis should be excluded in cases of colorectal carcinoma	Patient with colorectal cancer with two or more first-degree or second-degree relatives with a Lynch syndrome-related tumor, regardless of age

#### Phenotypic variation in LS

The clinical presentation of a patient with LS can vary depending on the MMR gene affected in the germline. Patients with *MLH1* mutations typically present with classic LS (CRC as the first presenting symptom with a mean age of 43 to 46). Similarly, patients with *MSH2* mutations also present with classic LS, however these patients are also at increased risk of extra-colonic cancers. Muir-Torre syndrome, which is a rare variant of LS in which patients develop hair follicle and sebaceous gland neoplasms, have *MSH2* germline mutations. Patients with germline mutations in *MSH6* are more likely to develop endometrial cancer and may not test positive for MSI by PCR. Mutations in PMS2 tend to develop CRC at an older age compared to classic LS. The rare biallelic mutation in any of the four MMR genes (constitutional mismatch repair deficiency syndrome) manifests as very early-onset (pediatric) hematological, colorectal, urinary tract and brain (glioblastoma) cancers, and neurofibromatosis [9].

### Unexplained dMMR colorectal cancers (sporadic dMMR-somatic MMR mutation and others)

This is an ill-defined group of MSI CRC patients who have discordant screening and germline results. In these cases, the initial screening for LS is positive (either abnormal MMR IHC or MSI PCR); however, *BRAF* V600E PCR and/or *MLH1* promoter hypermethylation studies does not indicate sporadic CRC, and genetic sequencing for MMR genes do not identify a germline mutation. These unexplained dMMR cases have been referred to as “Lynch-like syndrome” in the literature, however the causes for these cases are heterogenous and Lynch-like is a misleading nomenclature.

Recent studies have elucidated some of the causes/errors that could make a tumor look like a sporadic dMMR tumor. Approximately 70% of these patients may have biallelic somatic mutations in the MMR gene [20–22]. These patients with biallelic somatic mutations do not carry a germline mutation in MMR genes and therefore their progeny are not at risk of LS, and do not need lifelong intense screening protocol like LS patients.

Other potential explanations for unexplained dMMR cases [23] include: (a) incorrect performance/interpretation of MMR IHC results (absence of nuclear staining due to technical limitations or professional misinterpretations) (20%); (b) failure to detect germline *MMR* gene alteration using currently available tests (such as inversion of *MSH2* exons 1 to 7 that is not tested by many commercial labs) (unknown%); (c) other germline gene defects that also cause MSI phenotype, such as biallelic MUTYH mutation (3%) [24–26]; (d) somatic mosaicism (<1%) [27–29]; (e) constitutional epimutation of *MLH1* (germline monoallelic hypermethylation of the *MLH1* promoter region; no alteration to the genetic sequence of *MLH1*) (<1%) [30–32].

### Constitutional mismatch repair deficiency syndrome (CMMRD)

CMMRD patients have germline mutation of both alleles of a MMR gene (*MLH1, PMS2, MSH2, MSH6*), leading to loss of expression of the corresponding MMR protein. Unlike LS, CMMRD patients show loss of MMR staining in both the tumor and the background non-neoplastic tissue.

### Clinicopathologic features of MSI CRC

Clinically, tumors with MSI are more common in the right colon [33]. Sporadic tumors typically occur in older female patients, whereas, CRC in the context of LS often occurs in younger patients (50 years of age or less). These tumors respond poorly to 5-fluorouracil based chemotherapies [34–36], but patients with MSI tumors have a better prognosis than those with stage- matched MSS tumors [37–40].

Microscopically, MSI tumors share similar histomorphology regardless of their respective pathogenesis. They frequently have a mucinous phenotype, and are either very well-differentiated or poorly differentiated. The presence of signet ring cells or a medullary phenotype is also frequently seen in this context. Intratumoral heterogeneity (mixed conventional, mucinous, and poorly differentiated carcinoma) is common. The “dirty necrosis” seen in microsatellite-stable colon cancers is frequently absent. Tumors often contain increased intratumoral lymphocytes, and may have a Crohn’s -like reaction with prominent lymphoid aggregates at the periphery of the tumor [13, 41–43].

The histologic features of germline and sporadic MSI tumors are similar, although finding an adjacent sessile serrated adenoma as the precursor lesion is more suggestive of a sporadic tumor. In contrast, the precursor lesion associated with LS is a tubular adenoma. A recent study by Mas-Moya et al [44] demonstrated that when compared to LS, Lynch-like syndrome is more likely to occur in the right colon (a subset of LS tumors occurs at left colon [45]), more often identified in patients with tumors that harbor concurrent MLH1/PMS2 loss (with lack of MLH1 promoter hypermethylation) or concurrent MSH2/MSH6 loss, and less likely to have synchronous or metachronous LS-associated cancers.

The prognosis of patients with CRC associated with LS and *MLH1* promoter hypermethylation appear to be similar [46]. In up to 70% of CRC with hypermethylation of the *MLH1* promoter, there is a *BRAF* V600E gene mutation. CRC patients with *BRAF* V600E mutation have been shown to have a limited clinical response to epidermal growth factor receptor (EGFR) targeted therapies (cetuximab or panitumumab). Recent studies indicated that therapies targeting the PD-1 immune checkpoint blockade are effective in treating dMMR CRC [47].

### Screening for MSI and LS

Screening for MMR function can be done using IHC for the MMR proteins, and/or MSI analysis by polymerase chain reaction (PCR).

#### MMR protein expression by IHC

is performed to detect the absence or loss of a particular protein within the nucleus of the tumor cells; abnormal IHC correlates strongly with MSI status by PCR. MMR proteins are normally present in many human cells, particularly in proliferating cells such as crypt epithelium. Understanding the biochemistry/molecular biology of MMR proteins is important in the interpretation of IHC results. The most common mutations found in LS are frameshift or nonsense mutations that cause protein truncation or increased degradation. These mutations result in absence/loss of the mutated protein, which is easily detected by IHC. The MMR proteins MLH1, PMS2, MSH2 and MSH6 form heterodimer pairs in vivo (MLH1/PMS2 (MutLalpha) and MSH2/MSH6 (MutSalpha)). When either *MLH1* or *MSH2* is mutated and degraded in vivo, this will cause subsequent degradation and protein loss of its partner PMS2 or MSH6, respectively. For instance, when *MSH2* is mutated, this will appear immunohistochemically as absence/loss of MSH2 and MSH6. However, the opposite situation is not typically found. When either germline *PMS2* or *MSH6* is mutated and therefore degraded in vivo, there is usually no concomitant loss of protein expression of MLH1 or MSH2, respectively. This is due to the compensatory effects of other MMR proteins such as MSH3, which can bind to, and stabilize, either MLH1 or MSH2. Tumors that show absence of MSH2 and/or MSH6 or PMS2 are suspicious for LS, and these patients should be considered for sequencing of whatever protein was missing (after proper consent). If a germline mutation is then identified, this is diagnostic of LS. If a germline mutation is not identified, somatic MMR gene and/or loss of heterozygosity testing may be considered.

#### MSI by PCR

evaluates tumors by amplifying microsatellite repeats. The most widely used panel, the Bethesda panel, consists of five microsatellite repeats, including 2 mononucleotide repeats (BAT25 and BAT26) and 3 dinucleotide repeats (D2S123, D5S346, and D17S250) [48]. Microsatellites demonstrating at least two novel alleles (shift) define MSI high (considered to be microsatellite unstable). MSI low is defined as the detection of only one unique (shifted) microsatellite, although the significance of this category is controversial. When no shifted microsatellites are identified, this is defined as MSS. If MSI analysis is used as the initial screening test rather than IHC, *BRAF* mutational testing or *MLH1* promoter methylation can be used as the second step prior to genetic sequencing in patients found to have MSI.

Either MMR IHC or MSI by PCR works well for screening most cases, but each has limitations. IHC labs are much more widely available than molecular labs, but IHC results may be more affected by tissue fixation conditions. MSI analysis requires normal tissue in addition to tumor tissue for comparison, and may require tumor microdissection, but is not typically affected by fixation. Individual laboratories and multidisciplinary teams ultimately must determine for themselves which technique(s) is most effective in their institution. This issue may become less important with the advent of next generation sequencing panels [49], in which the cost for testing for all the MMR genes may in some laboratories be similar to testing for a one single MMR gene. Of note, MSI by PCR and MMR by IHC rarely may miss *MSH6* mutations, because *MSH6* mutations are often MSI-low or MSS. This is a known pitfall in MSI testing for gynecologic cancers given a larger proportion of endometrial cancers harbor *MSH6* mutations compared to CRC.

Approximately 5 to 10% of all CRC have substitution of valine with glutamic acid at amino acid position 600 of *BRAF*. BRAF is a protein in the mitogen active protein kinase pathway and acts as a serine/threonine kinase. The *BRAF V600E* mutation causes constitutive activation of the kinase and increases MAP kinase signaling. CRC with *BRAF* mutations are more commonly found in the right colon, have a high histologic grade, show female predominance, and more frequently originate from serrated polyps. Importantly, *BRAF V600E* mutations are almost never found in LS, but are seen in 40–76% of sporadic MSI CRCs. Therefore, in screening algorithms for patients with CRC, analysis of BRAF is a cost-effective method to screen for patients who should undergo further genetic testing for LS rather than gene sequencing in all those with absence of MLH1 and PMS2 [50, 51].

Besides being useful in distinguishing sporadic CRCs from LS, *BRAF V600E* also has prognostic value, as mutations have been suggested to be associated with a worse outcome in MSS CRC. It may also predict resistance to EGFR mediated antibodies [52]. Since *BRAF* and *KRAS* are part of the same MAP kinase pathway, it is not surprising that *BRAF* V600E mutations are almost mutually exclusive of *KRAS* mutations. Of note, *BRAF* mutation testing has no role in many extracolonic LS-associated cancers, including endometrial and ovarian cancers [53].

In patients with MLH1-deficient CRC, direct testing of *MLH1* promoter hypermethylation and/or *BRAF* V600E mutational analysis, may be a cost-effective means of identifying patients with sporadic tumors for whom germline genetic testing is not indicated.

**Fig. 1:**
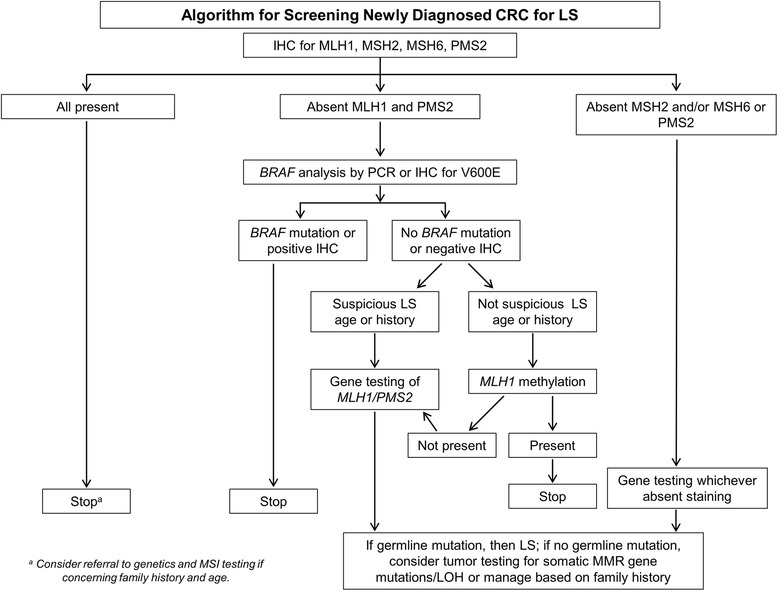
Algorithm for screening newly diagnosed colorectal carcinoma (CRC) for Lynch syndrome (LS). The initial step is to screen cases using immunohistochemistry (IHC) for mismatch repair proteins. When MLH1 and PMS2 are absent, the subsequent step is to utilize BRAF analysis by polymerase chain reaction (PCR) or immunohistochemistry

Figure 1 demonstrates the algorithm we currently use for screening newly diagnosed CRC for LS. Initially, the cancer is immunostained for MLH1, MSH2, MSH6, and PMS2. When all four proteins are present, no further work up is necessary unless there is specific clinical concern. When MLH1 and PMS2 are absent by IHC, the next step is analysis of *BRAF* by either PCR or IHC [50, 51, 54, 55] to help determine if the patient has a sporadic tumor or should be further evaluated for LS. We use PCR rather than IHC for BRAF. If *BRAF* V600E mutation is found, no further testing is required, as the tumor is presumed to be sporadic CRC. If no *BRAF* mutation is detected, the subsequent step depends on the degree of suspicion of LS based on the patient’s age and history. If there is suspicion of LS, the tumor will be sent for genetic sequencing of *MLH1* or *PMS2*. Conversely, if there is low suspicion of LS, *MLH1* promoter hypermethylation is then analyzed. If *MLH1* promoter hypermethylation is not detected, the patient will be sent for sequencing of *MLH1* or *PMS2*. Some laboratories use *MLH1* methylation testing rather than *BRAF* mutational analysis after IHC in those tumors found to have absence of MLH1 and PMS2. Similar to *BRAF* mutated cases, those with *MLH1* methylation are presumed to represent sporadic tumors and do not need to undergo further molecular testing for LS.

### Practical guide for MMR IHC interpretation

#### MMR sensitivity and specificity

MMR IHC has good sensitivity for MSI-high tumors (>90%) [56–59]; however it suffers lower sensitivity for MSI-low tumors. In terms of specificity, MMR IHC is efficient in detecting LS with *MSH2* and *MSH6* mutation; however specificity for *MLH1* and *PMS2* is low due to *MLH1* hypermethylation [60, 61]. MLH1 and PMS2 often show loss of expression in sporadic CRC due to transcriptional silencing of *MLH1* by promoter hypermethylation.

#### MMR staining patterns

Most cases screened with MMR IHC show typical patterns of staining and the interpretation is relatively straight forward. MMR proteins function in the cell nuclei, therefore the staining pattern is nuclear. However, punctate/speckled nuclear staining and nuclear membrane staining are considered abnormal staining patterns that should not be interpreted as preserved MMR protein expression [23]. They either represent loss of expression or staining artifact and therefore warrant additional investigation (compare staining to the internal control, repeat on a different block or sample, or MSI by PCR).

It is important to point out that it is common to see patchy (intratumoral heterogeneity) MMR staining in MSS tumors. Tissue hypoxia and fixation issues have been implicated for the staining heterogeneity [62, 63]. No definite consensus is present regarding the minimal percentage of positive nuclei to be present to indicate preserved/intact expression. In our practice, if more than 5% tumor nuclei show unequivocal nuclear staining (some use >10% [23], or any convincing staining [64]), it is considered normal/intact staining pattern. However, if less than but close to 5% tumor nuclei are positive, repeating the stain is the next step. If the repeat is similar, the stain result is considered equivocal and additional testing (MSI by PCR or genetic) is suggested. Of note, one needs to always make sure the positive internal control (stromal cells, lymphocytes, or nonneoplastic epithelial cells), shows nuclear positivity, before calling ‘loss of staining’ in the tumor cells.

It is advisable not to use ‘positive’ or ‘negative’ when reporting MMR IHC results. In the case of MMR IHC, positive (presence of) staining is a normal result, while negative (lack of) staining is abnormal and suggests the need for further testing. Therefore, the conventional “positive” and “negative” descriptors can be confusing and should be avoided in MMR IHC reports. We prefer to use the terminology “intact” when tumor nuclei show staining, and “loss” when tumor nuclei lack staining and this language is used in the College of American Pathologists (CAP) biomarker protocol [64].

**Fig. 2:**
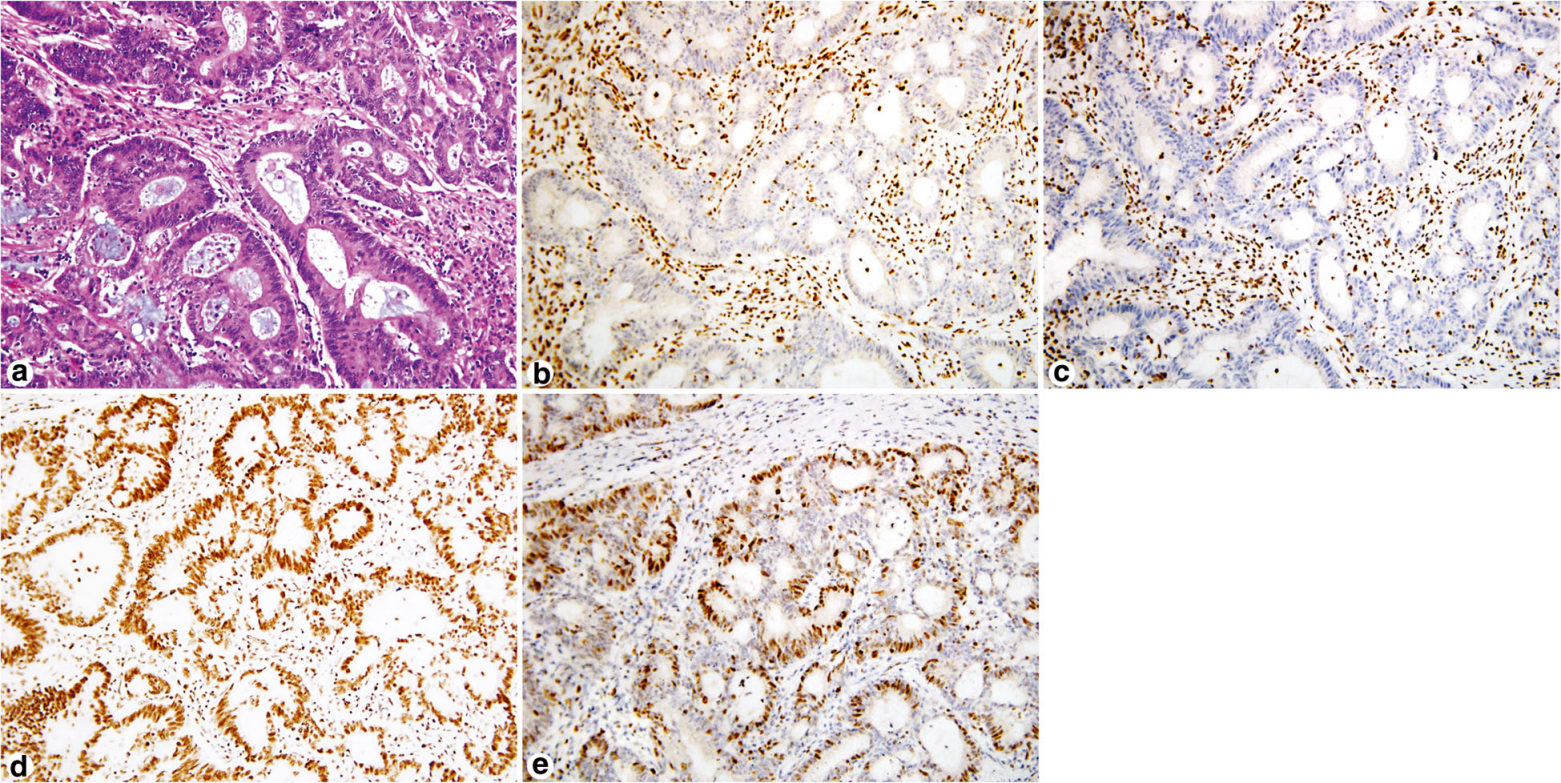
Immunohistochemistry for mismatch repair proteins in a patient with sporadic hypermethylation of the MLH1 promoter. Hematoxylin & eosin (H&E) stain of the adenocarcinoma (**a**). Tumor cells show loss of MLH1 nuclear expression (**b**) and loss of PMS2 nuclear expression (**c**) while the stroma and lymphocytes shows strong intact staining. Conversely, tumor cells show intact nuclear expression of MSH2 (**d**) and MSH6 (**e**). This tumor showed *BRAF* V600E mutation by PCR consistent with sporadic microsatellite-unstable colorectal carcinoma

**Fig. 3:**
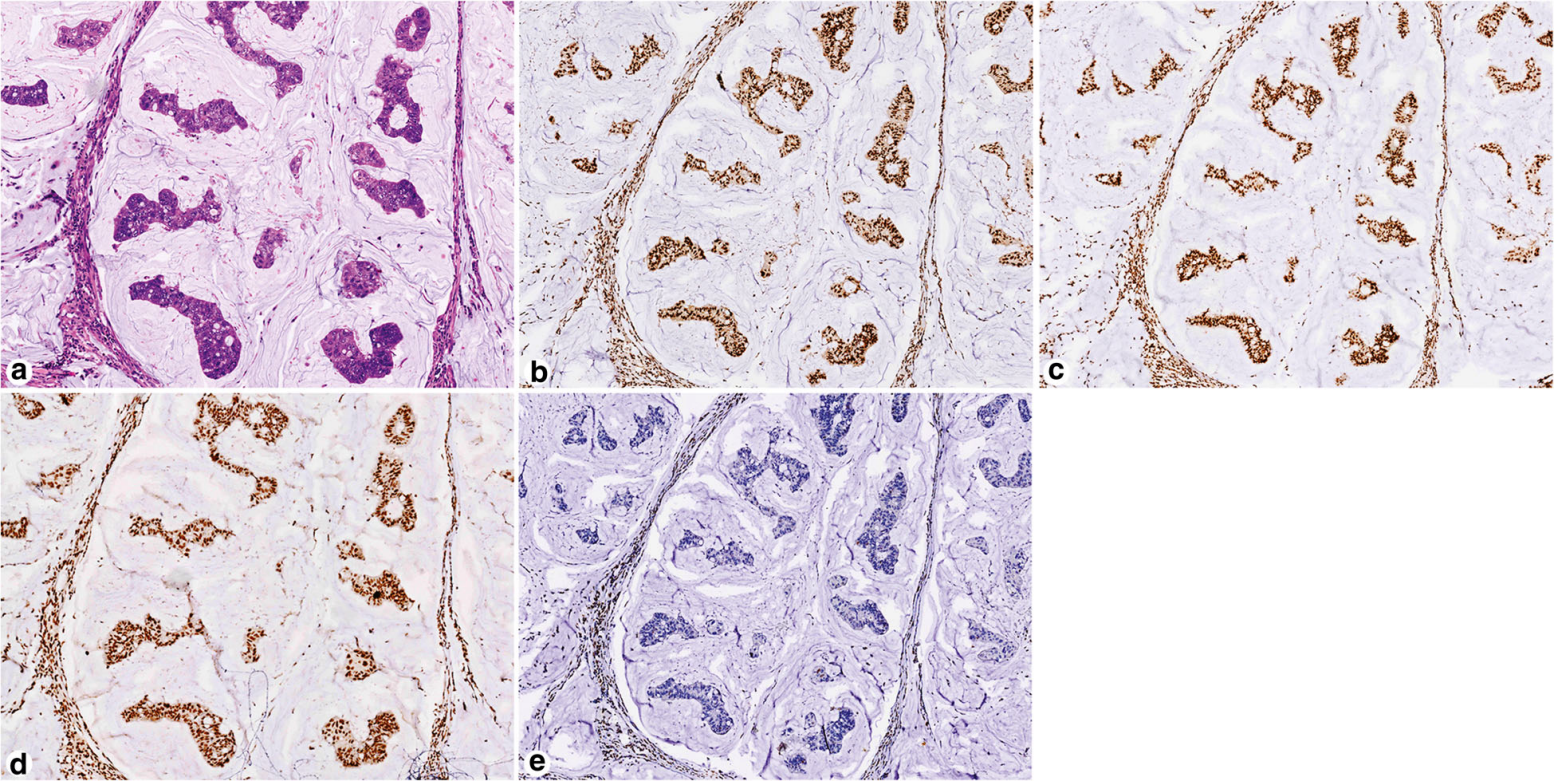
Immunohistochemistry for mismatch repair proteins in a patient with Lynch syndrome. H&E stain of the mucinous adenocarcinoma (**a**). Intact nuclear expression of MLH1 (**b**) and PMS2 (**c**). Intact nuclear expression of MSH2 (**d**). This tumor showed absent nuclear staining of MSH6 (**e**). Genetic sequencing confirmed mutation of the *MSH6* gene

As discussed previously, the pairing and dimerization of the MMR proteins stabilize the function unit, which help to explain the staining pattern of MMR expression/loss observed. Figures 2 and 3 demonstrate two cases with abnormal MMR IHC results. Figure 2 demonstrates MMR IHC in a patient with sporadic hypermethylation of the MLH1 promoter, in which both MLH1 and PMS2 show loss of expression. Figure 3 demonstrates MMR IHC in a patient with LS with isolated loss of MSH6.

When all four MMR proteins are absent, the possibility of poor fixation and loss of antigenicity should be considered. The surrounding stroma is an excellent positive control and should always show staining. On the other hand, intact expression of all four proteins does not exclude LS. About 5% of families harbor a missense mutation (most commonly in *MLH1*) that results in a nonfunctional protein with retained antigenicity.

Unusual Patterns That Can Lead to Confusion

- In cases of isolated loss of PMS2 IHC, germline *MLH1* analysis should be performed if no mutations are detected through *PMS2* testing. In a study by Dudley et al [65], approximately 24% of patient with tumors with isolated loss of PMS2 expression harbor germline *MLH1* mutation. Furthermore, a subset of *MLH1* mutations result in functionally inactive MLH1 protein that is antigenically intact and will be detected by the commonly used anti-MLH1 antibody clones. Such germline *MLH1* mutations will lead to decreased MLH1 protein stability and/or quantity, compromised stability of MLH1-PMS2 complexes, and subsequent PMS2 degradation. Recently, Rosty et al. [66] demonstrated similar findings, and they also recommend germline *MLH1* mutation analysis in individuals with isolated loss of PMS2 expression but without *PMS2* mutation identified.
- Very rarely, all four stains are lost (null pattern) due to a germline *MSH2* mutation (causing absent MSH2/MSH6) together with a somatic *MLH1* hypermethylation (causing absent MLH1/PMS2) [67].
- Another uncommon finding is loss of MSH6 together with MLH1 and PMS2. This could occur due to *MLH1* promoter hypermethylation causing MLH1/PMS2 loss, leading to MSI. Such *MLH1/PMS2* deficient, MSI tumors, are prone to generate somatic mutations in *MSH6* gene, which cause significantly reduced staining of MSH6 [68].

#### FAQs for MMR immunohistochemistry

1. A patient’s colon cancer was previously screened for LS and no abnormality was found, should I screen a second colon or endometrial cancer in this patient?
  Answer: MMR IHC of synchronous/metachronous neoplasms in LS patients demonstrated discordant MMR immunoreactivity in 31% cases [69], therefore it may be worthwhile to perform LS screening in all primary, synchronous, and metachronous LS-associated neoplasms if a previous tumor screened intact.
2. A Gastroenterologist asked to test MMR IHC in an adenoma, how should I proceed for the IHC interpretation?
  Answer: The criteria for interpreting MMR IHC in an adenoma are the same as for cancer. It has been shown that 50% to 72% of conventional adenomas from mutation carriers show loss of MMR protein expression concordant with the underlying germline mutation [70, 71]. In addition, the absence of staining was particularly frequent in larger adenomas in LS patients, was associated with adenomas with villous component, and was observed more often in adenoma with high-grade dysplasia. However, some adenomas in LS patients only contain loss of one allele and do not contain the second hit; and therefore still demonstrate preserved MMR protein expression. In signing out these cases we comment that “whereas absent staining may be seen in LS and the pattern of loss useful in directing gene testing, intact expression does not exclude the diagnosis.”
3. Does neoadjuvant therapy in rectal cancer affect MMR IHC result?
  Answer: Rectal tumors treated with neoadjuvant therapy can sometimes show decreased or complete loss of MSH6 staining or only a nucleolar staining pattern (Fig. 4) [72, 73]. Evidence has shown that most of these cases do not have a *MSH6* mutation and this should not be interpreted as loss/absence of MSH6. Rather than immediately sequencing questionable cases, repeating the IHC or performing IHC on the pretreatment biopsy will help resolve this issue. Decreased expression of PMS2 has also been reported in 30% of posttreatment rectal carcinomas [74]

**Fig. 4:**
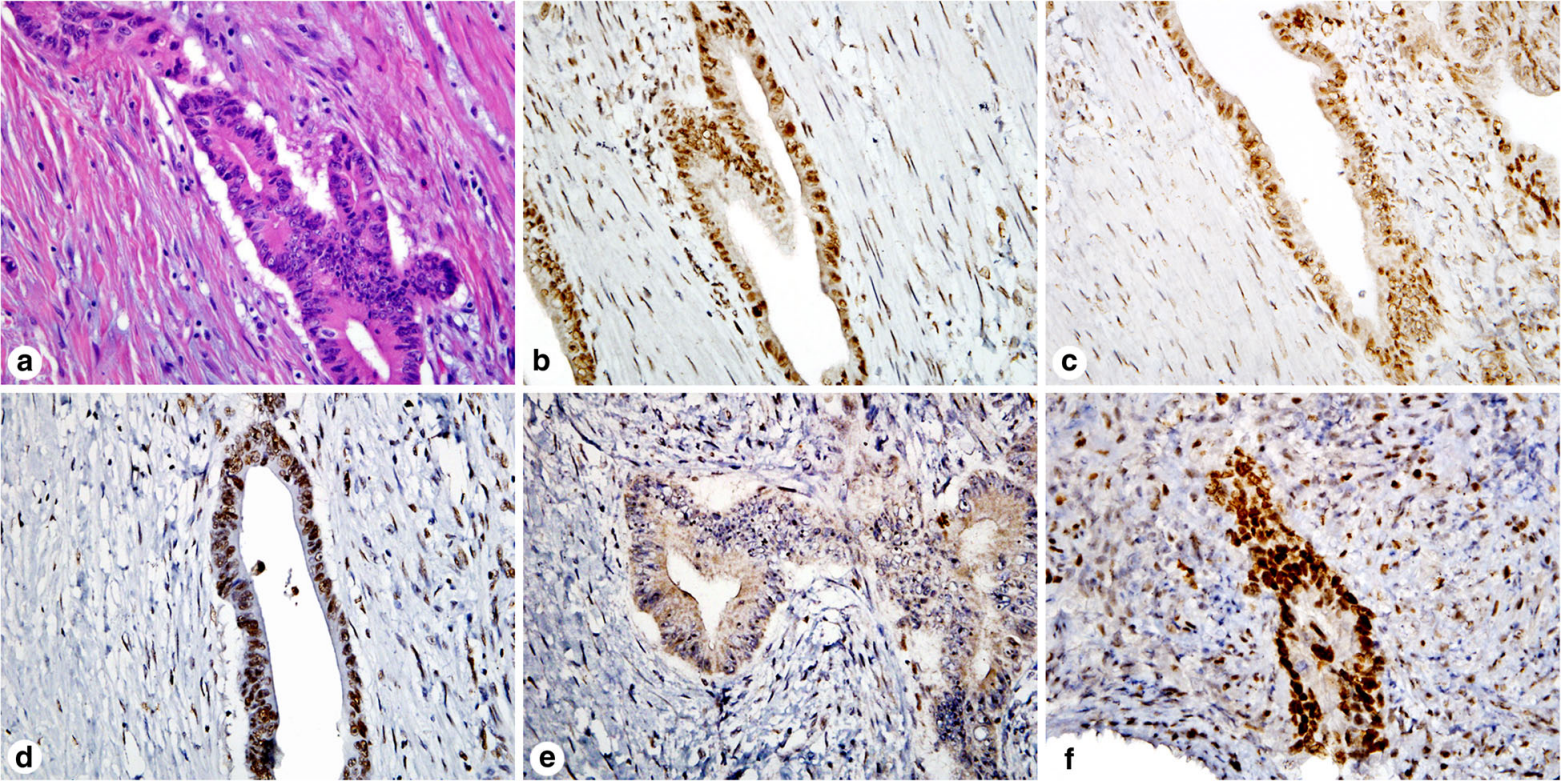
Immunohistochemistry for mismatch repair proteins in a patient that received neoadjuvant chemotherapy for rectal adenocarcinoma. H&E stain of the tumor in the resection specimen (**a**). The resection specimen showed intact MLH1 (**b**), PMS2 (**c**), and MSH2 (**d**) staining. MSH6 staining of the resection specimen showed focal nucleolar staining (**e**) that was originally interpreted as absent, but subsequent molecular sequencing did not reveal a mutation. The pretreatment tumor biopsy was stained for MSH6 and showed intact staining (**f**)
4. Can MMR IHC be performed on autopsy cases?
  Answer: Yes, MMR IHC may be performed on autopsy cases, but be aware of the inferior preservation of the tumor tissue in autopsy cases, which may lead to weak and patchy nuclear staining of the tumor cells. Nonetheless, if the internal control (adjacent benign epithelium, stroma or background lymphocytes) demonstrates similar weak staining, it is most likely that the MMR protein expression is intact/normal.
5. Can MMR IHC be performed on metastasis?
  Answer: Our group recently demonstrated [75] that in 50 primary CRC with metastasis, there is 100% concordance of MMR IHC results between primary and metastasis, suggesting that using metastatic tissue to identify patients with dMMR tumors (screen for LS or to aid in therapeutic choices) is feasible when the primary tissue is not available for testing.
6. Should MMR IHC be performed on serrated polyps to help exclude LS?
  Answer: No. CRC arising from the serrated pathway has different underlying molecular genetics than CRC arising from LS [76], although both may show dMMR. The genetic signature of CRC arising from serrated polyps often contains *BRAF* mutation and *MLH1* promoter hypermethylation. In contrast, LS almost never harbors *BRAF* mutation.

## Conclusions

Detecting LS is desirable because intensified clinical cancer surveillance saves lives. Identification of MSI CRC is important, as dMMR may serve as a screening tool for detecting LS, a prognostic marker for patient outcome, and a predictive marker for response to chemotherapy. Tumor DNA sequencing should be undertaken in unsolved cases of abnormal LS screening without identifiable germline mutation, to evaluate for somatic biallelic mutations, as it can explain two thirds of these cases and help guide genetic counseling and reduce patient anxiety. Next generation sequencing methodology has been employed for detection of molecular alterations in dMMR CRC and may replace the current method of detecting MSI by PCR techniques in the near future.

## Abbreviations

CIMP: CpG Island Methylator Phenotype
dMMR: Deficient DNA mismatch repair
HNPCC: Hereditary Nonpolyposis Colorectal Cancer
IHC: Immunohistochemistry
LS: Lynch syndrome
MMR: Mismatch repair
MSI: Microsatellite instability
MSS: Microsatellite stability
PCR: Polymerase chain reaction
